# Adjustment of serum HE4 to reduced glomerular filtration and its use in biomarker-based prediction of deep myometrial invasion in endometrial cancer

**DOI:** 10.18632/oncotarget.22599

**Published:** 2017-11-21

**Authors:** Josef Chovanec, Iveta Selingerova, Kristina Greplova, Sofie Leisby Antonsen, Monika Nalezinska, Claus Høgdall, Estrid Høgdall, Erik Søgaard-Andersen, Kirsten M. Jochumsen, Pavel Fabian, Dalibor Valik, Lenka Zdrazilova-Dubska

**Affiliations:** ^1^ Clinic of Surgical Oncology, Masaryk Memorial Cancer Institute, Brno, Czech Republic; ^2^ Regional Centre of Applied Molecular Oncology, Masaryk Memorial Cancer Institute, Brno, Czech Republic; ^3^ Department of Laboratory Medicine, Masaryk Memorial Cancer Institute, Brno, Czech Republic; ^4^ Gynecologic Clinic, Rigshospitalet, Copenhagen University Hospital, Copenhagen, Denmark; ^5^ Department of Pathology, Danish Cancer Biobank, Herlev University Hospital, Herlev, Denmark; ^6^ Department of Gynecology and Obstetrics, Aalborg University Hospital, Aalborg, Denmark; ^7^ Department of Gynecology and Obstetrics, Odense University Hospital, Odense, Denmark; ^8^ Department of Oncological Pathology, Masaryk Memorial Cancer Institute, Brno, Czech Republic

**Keywords:** predictive biomarkers, HE4, glomerular filtration rate, endometrial cancer, deep myometrial invasion

## Abstract

**Background:**

We investigated the efficacy of circulating biomarkers together with histological grade and age to predict deep myometrial invasion (dMI) in endometrial cancer patients.

**Methods:**

HE4ren was developed adjusting HE4 serum levels towards decreased glomerular filtration rate as quantified by the eGFR-EPI formula. Preoperative HE4, HE4ren, CA125, age, and grade were evaluated in the context of perioperative depth of myometrial invasion in endometrial cancer (EC) patients. Continuous and categorized models were developed by binary logistic regression for any-grade and for G1-or-G2 patients based on single-institution data from 120 EC patients and validated against multicentric data from 379 EC patients.

**Results:**

In non-cancer individuals, serum HE4 levels increase log-linearly with reduced glomerular filtration of eGFR ≤ 90 ml/min/1.73 m^2^. HE4ren, adjusting HE4 serum levels to decreased eGFR, was calculated as follows: HE4ren = exp[ln(HE4) + 2.182 × (eGFR-90) × 10^-2^]. Serum HE4 but not HE4ren is correlated with age. Model with continuous HE4ren, age, and grade predicted dMI in G1-or-G2 EC patients with AUC = 0.833 and AUC = 0.715, respectively, in two validation sets. In a simplified categorical model for G1-or-G2 patients, risk factors were determined as grade 2, HE4ren ≥ 45 pmol/l, CA125 ≥ 35 U/ml, and age ≥ 60. Cumulation of weighted risk factors enabled classification of EC patients to low-risk or high-risk for dMI.

**Conclusions:**

We have introduced the HE4ren formula, adjusting serum HE4 levels to reduced eGFR that enables quantification of time-dependent changes in HE4 production and elimination irrespective of age and renal function in women. Utilizing HE4ren improves performance of biomarker-based models for prediction of dMI in endometrial cancer patients.

## INTRODUCTION

Endometrial cancer (EC) is a malignant tumor of endometrial epithelial origin, accounting for 20% to 30% of malignant diseases of the female reproductive system [1]. With early manifestation by abnormal vaginal bleeding after menopause, approximately 70% of endometrial cancers are diagnosed at early stages [2]. Prognosis of early stage endometrial carcinoma is relatively good in comparison with other gynecological malignancies. However, the remaining 30% of patients are diagnosed with endometrial cancer at advanced stages. Beside FIGO status, the main prognostic factors include: histological type (endometrioid versus clear cell, squamous, mucinous, serous or mixed types), grade, level of myometrial invasion (MI) and the status of pelvic and paraaortal lymphatic nodes.

Based on FIGO criteria, endometrial cancer is surgically staged [3] by a procedure that may include hysterectomy, bilateral salpingo-oophorectomy, a collection of pelvic washings, and a selected pelvic and para-aortic lymph node dissection. Owing to potential morbidity associated with lymphadenectomy leading to symptomatic lymphocysts, lymphedema, deep vein thrombosis, neurologic injury, vein injury, and need for blood transfusion, the decision regarding lymph node dissection should be based on quantification of risk factors for nodal metastasis, such as histology of tumor, grade, and myometrial invasion ≥ 50% (deep MI, dMI) [4]. The depth of myometrial spreading is closely related to lymph node invasion and correlates with overall survival of patients [5]. Due to the need to plan where to perform the surgical procedure (in general gynecological centres versus tertiary gyneco-oncology centres), and the surgical procedure itself (laparoscopy versus open surgery with respect to maximizing clinical benefits), various preoperative imaging modalities and histological examinations of biopsies are exploited with variable predictive value towards dMI [6–8]. While for high-risk patients with grade 3 endometrial cancer, lymphadenectomy should be recommended [9] the risk stratification for grade 1 or 2 EC patients can be done perioperatively based on the depth of myometrial invasion [9, 10]. Therefore, the inclusion of preoperative levels of peripheral blood-derived biomarkers is potentially informative when assessing dMI and is highly relevant for the risk prediction and decision making process for the upfront assessment of the extent of endometrial cancer surgery [11, 12].

CA125 is elevated in both primary and recurrent endometrial cancers [13–15] and has been shown to be predictive of extrauterine disease [16]. The predictive value of CA125 is, however, limited due to its low specificity resulting from CA125 elevation in a variety of benign conditions [17–20]. Human epididymis protein 4 (HE4) was shown to be overexpressed in serous and endometrioid epithelial ovarian carcinomas and uterine cancers and to a lesser extent in pulmonary, breast, gastrointestinal, and urological carcinomas [21] and is often detectable in the bloodstream of cancer patients including patients with endometrial carcinoma [22–25]. Unlike CA125 [26], HE4 is cleared from the systemic circulation through glomerular filtration due to its small molecular size [27]. Consequently, the informative relevance of HE4 is hampered in individuals with impaired glomerular functions, with false positive levels within the range 500-1000 pmol/l in patients with chronic kidney disease (CKD) degree 4-5 [28, 29]. Thus, decreased renal function needs to be considered in the interpretation of HE4 levels with greater importance in endometrial cancer patients, where the elevation of serum HE4 is often subtle compared to ovarian cancer patients.

The aim of this multicentre international study is to evaluate preoperative circulating biomarkers CA125, HE4, and HE4ren (HE4 level adjusted to glomerular filtration rate), together with histological grade and age in predictive models of the depth of myometrial invasion in endometrial cancer patients to i) identify cases that would benefit from lymphadenectomy and ii) exclude from extensive surgery those that would not.

## RESULTS

### Serum HE4 levels are increased by impaired renal function: calculation of HE4ren by adjusting serum HE4 to eGFR

**Figure 1:**
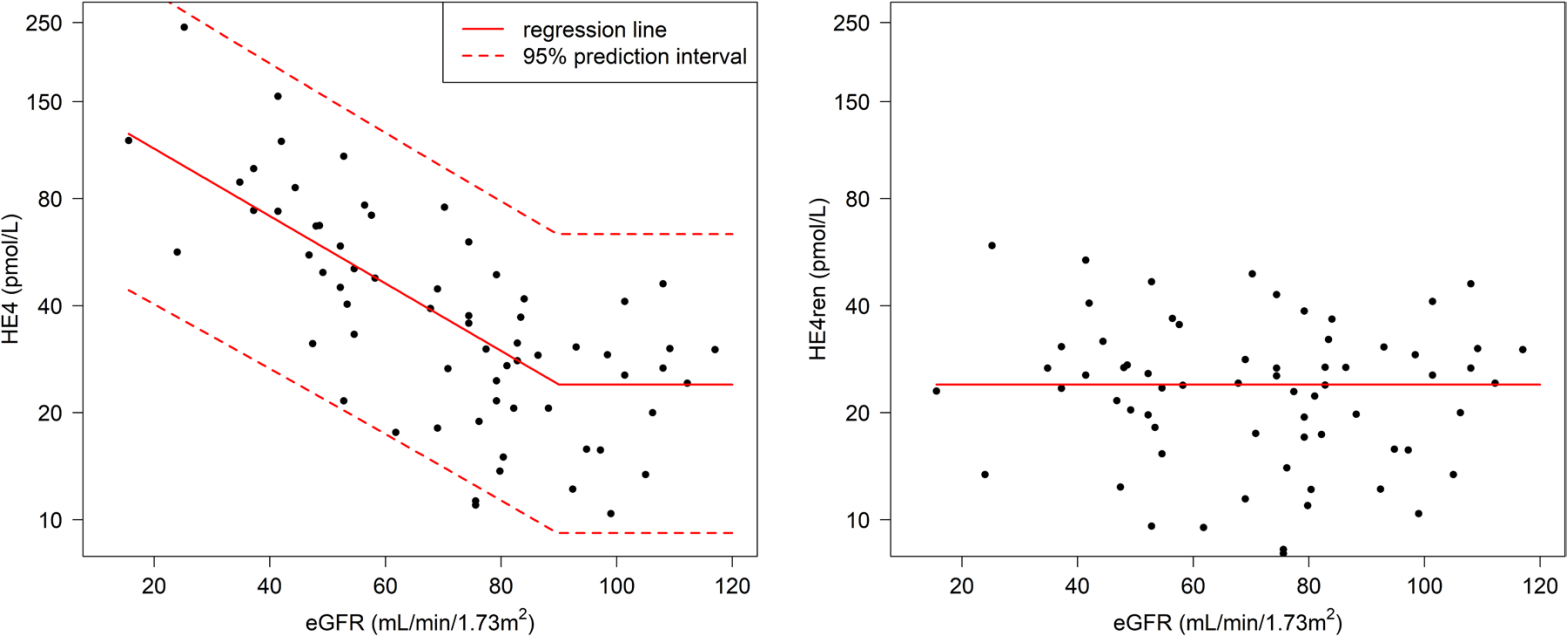
Relationship between HE4 measurements and eGFR Left panel: Association between serum HE4 level (pmol/l) and eGFR (ml/min/1.73 m^2^). Right panel: HE4ren was calculated to quantify serum HE4 in the range of decreased eGFR.

Serum HE4 levels were measured in a pool of gynecologically and oncologically healthy controls with normal or reduced kidney function as assessed by creatinine-based eGFR. In the range of eGFR ≤ 90 ml/min/1.73 m^2^, serum HE4 levels increased log-linearly (Figure 1 left). To adjust HE4 serum levels to decreased eGFR, a HE4ren was calculated as follows: HE4ren = exp[ln(HE4) + 2.182 × (eGFR-90) × 10^-2^] for the range eGFR ≤ 90 ml/ min/1.73 m^2^ ; HE4ren is equal to HE4 for the range eGFR > 90 ml/min/1.73 m^2^ (Figure 1 right). While HE4 is falsely elevated in individuals with impaired glomerular functions, the 95^th^ percentile of the HE4ren distribution in the CKD-positive control cohort corresponded to 46.6 pmol/l (Supplementary Figure 1).

**Figure 2:**
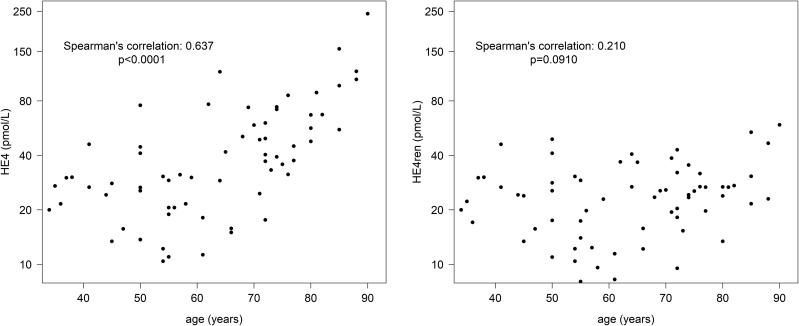
Relationship between HE4 measurements and age Left panel: Association between serum HE4 level (pmol/l) and age. Right panel: HE4ren is age-independent.

To address the issue of HE4 age-dependence, HE4 and HE4ren were evaluated in the context of age in controls, with results showing that serum HE4 was correlated with age (Spearman correlation coefficient 0.637, p < 0.0001) (Figure 2 left). Applying HE4ren, the age-dependence was obviated (Spearman correlation coefficient 0.210, p = 0.0910) (Figure 2 right). Moreover, unlike HE4, HE4ren was independent of menopausal status in control group (Supplementary Figure 2).

### HE4ren improves the value of preoperative prediction of deep myometrial invasion in endometrial cancer patients

HE4ren was calculated for all patients in training and validation sets (Supplementary Table 1) and further evaluated together with other variables (CA125, grade, and age) to predict the dMI in EC patients. Univariable analysis of the predictive value of age, grade, CA125, HE4, and HE4ren was performed for 3 pools of patients (MMCI training, MMCI validation, ENDOMET validation) (Supplementary Table 2). As preoperative grade 3 endometrial cancer patients are managed as a high-risk group with open surgery, the univariable analysis was performed separately for the cohort of grade 1 plus grade 2 (G1-or-G2) patients (Supplementary Table 2).

Univariable analysis of the MMCI training set revealed that the following parameters are predictive of dMI: age, CA125, HE4 and HE4ren with best discriminative value achieved by HE4 with an AUC = 0.7951 for the any-grade pool of patients and AUC = 0.7912 for the grade 1-or-2 subcohort. In contrast, histological grade was not a statistically significant predictor of dMI (Supplementary Table 2).

### Continuous model development and validation

Four models based on continuous variables (age, CA125, grade, and HE4 or HE4ren) were developed by binary logistic regression on the training dataset (Table 1, Supplementary Table 3). In models for the any-grade cohort, grade and preoperative serum CA125 levels appeared to be insignificant predictors of dMI. Both HE4 and HE4ren were stronger predictors of dMI than age. In models for the G1-or-G2 subcohort, age, grade (G1 versus G2) and HE4 concentrations appeared to be significant predictors of dMI with HE4ren having the strongest predictive value (Table 1). In both validation sets, for the any-grade and G1-or-G2 subcohorts, the predictive value of models with continuous variables for dMI was higher for HE4ren-based models compared to HE4-based models.

**Table 1:** Multivariable analysis of dMI prediction with continuous variables

Model	Predictor					AUC		
	Age	HE4	HE4ren	Grade	CA125	MMCI training	MMCI validation	ENDOMET validation
**Any grade**								
Continuous-all-HE4	OR: 1.06 (1.01-1.13)p = 0.0355	OR: 5.80 (2.48-16.1)p = 0.0002		NS	NS	0.8045	0.7546	0.7043
Continuous-all-HE4ren	OR: 1.09 (1.04-1.16)p = 0.0020		OR: 6.66 (2.86-18.5)p < 0.0001	NS	NS	0.8221	0.7546	0.7125
**G1-or-G2**								
Continuous-G1/2-HE4	OR: 1.07 (1.00-1.14)p = 0.0587	OR: 4.84 (1.98-15.3)p = 0.0026		OR: 2.32 (0.89-6.33)p = 0.0899	NS	0.8162	0.7955	0.7043
**Continuous-G1/2-HE4ren**	**OR: 1.10 (1.03-1.18)p = 0.0051**		**OR: 8.08 (2.88-30.1)p = 0.0005**	**OR: 2.38 (0.88-6.74)p = 0.0913**	**NS**	**0.8373**	**0.8333**	**0.7146**
$P(dMI)=\frac{1}{1+e^{15.2-0.1age-2.1\log HE4ren-0.9(grade-1)}}$								

NS = not significant. Biomarker values were log (ln) transformed. The final model with real-life clinical applicability is shown in bold with detailed description of the model where *P(dMI)* stands for the probability of having myometrial invasion ≥ 50%.

**Figure 3:**
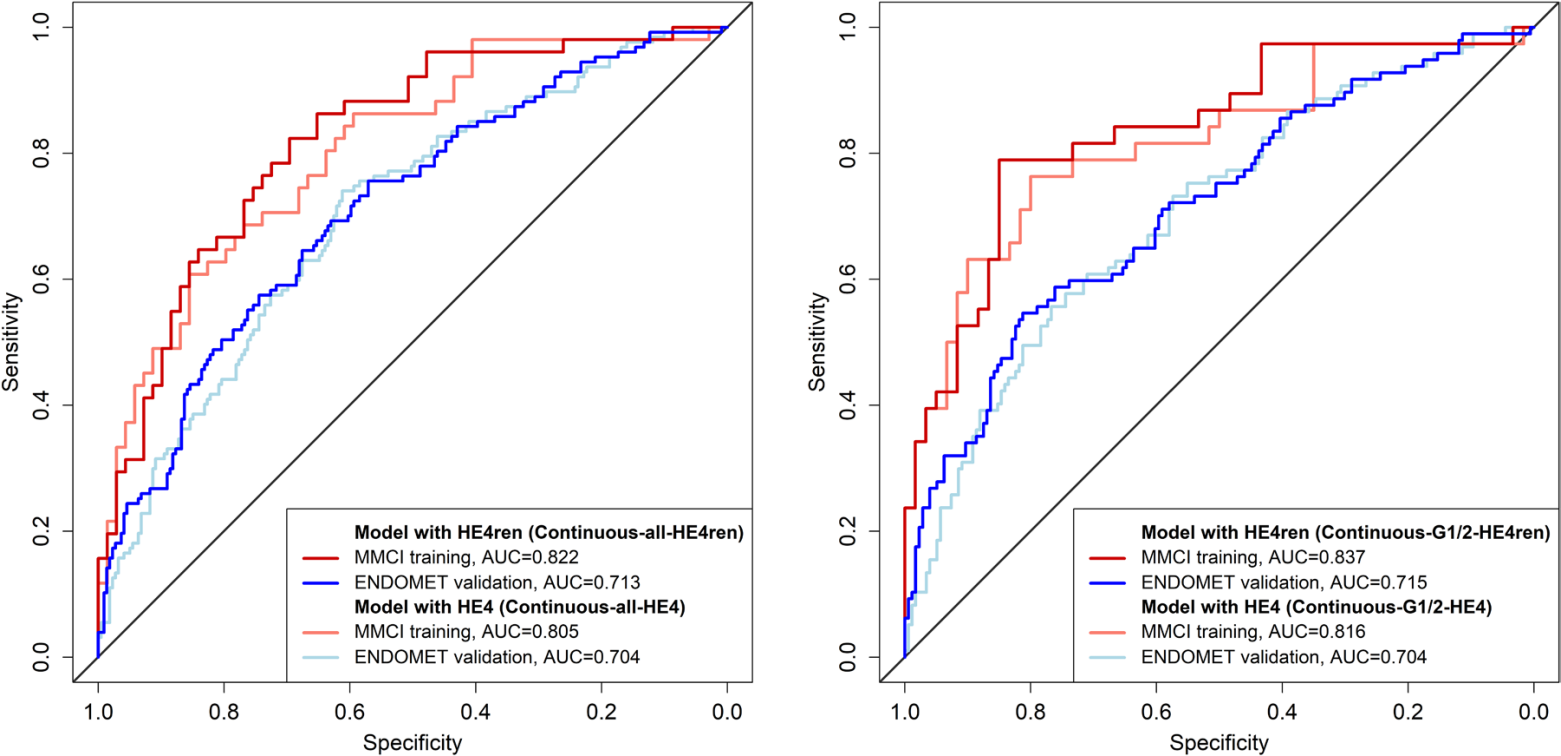
ROC curves of computational models with continuous variables

Application of the HE4ren-based final model on the G1-or-G2 MMCI validation set and ENDOMET validation set revealed an AUC = 0.8333 for MMCI validation set and AUC = 0.7146 for the ENDOMET validation set (Table 1). In the G1-or-G2 subcohort, the HE4ren-based model provided an improvement in dMI prediction compared to the HE4-based model in both the training set and the ENDOMET validation set (Figure 3).

The predictive values of the respective models in the training set and validation sets were calculated for various cut-off values of dMI probability (Supplementary Table 4). Optimal cut-off value of probability of dMI derived from the training dataset was 43% with sensitivity 78.9%, specificity 85.0%, positive predictive value 76.9% and negative predictive value 86.4%. This cut-off value of 43% yielded the most effective analytical parameters for both validation datasets: MMCI validation with sensitivity 66.7%, specificity 86.4%, positive predictive value 57.1% and negative predictive value 90.5%; ENDOMET validation with sensitivity 59.8%, specificity 70.5%, positive predictive value 52.7% and negative predictive value 76.1% (Supplementary Table 4).

### Categorical model development and validation

Models for categorical variables based on age, HE4ren, CA125, and grade were developed for any-grade conditions and G1-or-G2 conditions. In any-grade EC patients, age, HE4ren, and CA125 but not grade were significant predictors of dMI. In G1-or-G2 EC patients, all variables were predictors of dMI in this order of significance: HE4ren, age, CA125, histological grade (Supplementary Table 5). AUC values for training and validation datasets are shown in Supplementary Table 5.

For G1-or-G2 EC patients, specific score values for each categorical variable and total score for various levels of probability of dMI were calculated (Supplementary Table 5). The predictive value of the model in training set and validation sets were calculated for various cut-off values of dMI (Supplementary Table 6). With respect to highest sum of specificity and sensitivity, the optimal cut-off value derived from the training dataset was 33% (Supplementary Table 6). Testing these cut-off values in validation sets while favouring sensitivity, we observed the most effective analytical parameters for the 25% cut-off for both validation datasets: MMCI validation with positive predictive value 44.4% and negative predictive value 89.5%; ENDOMET validation with positive predictive value 51.4% and negative predictive value 82.7% (Supplementary Table 6). Twenty-five percent probability of having dMI was set as a cut-off between the “low-risk” and the “high-risk” groups of patients and enabled simplification of the model in the following way. A probability of dMI equal to 25% corresponded to total score 27.1. Grade was considered a “minor risk factor” with a simplified score value of 1, increased HE4ren and CA125 as an “intermediate risk factor” with simplified score value of 2, and age equal to or above 60 years as a “major risk factor” with a simplified score value of 3. A sum of these score values ≤ 3 corresponded to a probability less than 25% of having dMI and patients were thus classified as low-risk for dMI. A sum of these score values ≥ 4 corresponded to a probability above 25% of having dMI and patients were classified as high-risk for dMI (Supplementary Table 5). An algorithm tree for simplified dMI predictive model in G1/2 EC patients have been prepared (Supplementary Figure 3).

## DISCUSSION

We investigated the efficacy of circulating biomarkers to predict deep myometrial invasion in endometrial cancer patients. Preoperative stratification of the patients to high-risk or low-risk category may guide gynecologic oncology surgeons to select the patients that would benefit from extensive surgery including lymphadenectomy and consequently to plan the surgical procedure to the respective center.

Previous studies have shown that HE4 is a useful biomarker for stratifying endometrial cancer patients according to extent of disease [11, 30–34]. However, serum biomarker HE4 generally suffers from low specificity as shown by its accumulation in blood in patients with reduced glomerular functions [29, 35, 36]. Subsequently, there is no generally accepted cut-off value of HE4 for its primary clinical application in the diagnosis of ovarian cancer [36, 37]. In this study, we show that being elevated in subclinically reduced eGFR lower than 90 ml/min/1.73 m^2^, HE4 serum levels are highly sensitive to impaired glomerular filtration. eGFR-dependent levels of serum HE4 enabled us to develop the HE4ren formula that is suitable for quantifying serum HE4 concentrations in women with impaired renal function. Furthermore, here we show that HE4ren is independent of age and menopausal status suggesting that previously described age-associated decrease in glomerular filtration is an underlying factor for elevated serum HE4 levels in postmenopausal elderly women [36, 38, 39]. The 95^th^ percentile of HE4ren levels in control CKD-positive individuals corresponds to 46.6 pmol/l, suggesting that a cut-off value for HE4ren would be approximately 50 pmol/l irrespective of age. It is of note that these data and subsequent models are derived from Abbott-based serum HE4 and CA125 concentrations and eGFR based on a CKD-EPI equation employing creatinine quantified by enzymatic method. Unlike HE4 that is consistently and substantially higher in Li-heparin plasma compared to serum (data not shown), serum and plasma yields equal concentrations of creatinine and CA125. Therefore if single one blood specimen is preferred for the biomarker-based predictive evaluation of dMI in EC patients suggested here, all measurements should be performed from serum.

Reflecting the issue of CKD-induced increase of serum HE4 levels, Kappelmayer et al. proposed an algorithm for the prediction of ovarian cancer that uses eGFR, serum HE4, and CA125 levels - regardless of the menopause status - in patients who suffer from kidney disorders [35]. Compared to this algorithm, HE4ren provides the capability of longitudinal quantification of HE4 release to serum in conditions of varying severity of kidney impairment. Therefore, at MMCI, HE4ren is calculated and reported together with direct serum HE4 and CA125 concentrations to aid oncogynecologists in the management of the diagnostic process and the follow-up of ovarian cancer patients with CKD. The reporting of HE4 serum concentrations in the form of HE4ren during follow-up of ovarian cancer patients may augment the clinical validity of this biomarker as it takes into consideration invariable alteration of renal function due to nephrotoxic platinum-based treatment in ovarian cancer management. It remains to be investigated whether adjustment of HE4 to kidney insufficiency improves the predictive value of indexes that have been established to triage patients with suspected epithelial ovarian malignancies [40, 41].

In the evaluation of the continuous parameters HE4, CA125, HE4ren, age and grade in the preoperative prediction of dMI in endometrial cancer patients, direct HE4 has shown the strongest predictive power in the univariable analysis due to its dependence on age that is itself a strong predictor of dMI. On the other hand, age-dependence is eliminated in HE4ren. Testing models based on continuous variables to predict dMI, models with HE4ren yielded better analytical value for both any-grade patients and grade 1-or-2 patients compared to models with crude serum HE4 levels. As grade 3 endometrial cancer patients are considered and subsequently managed as high-risk, our final predictive models were set for grade 1-or-2 EC patients. We present the model based on continuous age, HE4ren and grade, calculating the probability of dMI in EC patients. A final simplified model with categorical variables based on the variables HE4ren, age, CA125, and grade, in this order of significance, was able to predict dMI with sensitivity of 90% and specificity of 57% in the training set. Applying a simplified categorical model on both validation datasets, the sensitivity was 67% and the specificity was 77% for the MMCI validation dataset and 60% sensitivity and 83% specificity was observed for the ENDOMET validation dataset. Applying newly developed HE4ren-based models on variables retrieved from the Danish ENDOMET project and their comparison with the original predictive model based on HE4 and CA125 in the ENDOMET study [11] revealed improvement in analytical values. Unlike for MMCI patients, elevated serum CA125 was a strong predictor of dMI in the ENDOMET validation dataset and thus the simplified categorical model yielded better analytical performance in the ENDOMET dataset than the continuous model due to an inclusion of CA125 into the categorical model.

In conclusion, we have introduced HE4ren adjusting serum HE4 levels to reduced glomerular filtration in women, enabling quantification of the dynamics of HE4 production irrespective of age and in conditions of impaired glomerular functions. Applying HE4ren improves the performance of developed models for prediction of dMI in endometrial cancer patients.

## MATERIALS AND METHODS

### Study design

First, a mathematical formula named “HE4ren” was developed with adjustment of HE4 serum levels towards decreased glomerular filtration rate in non-cancer individuals. Second, preoperative laboratory parameters HE4, HE4ren, CA125 and clinical parameters such as age and grade were evaluated to predict the preoperative risk of the depth of myometrial invasion in endometrial cancer patients. Continuous and categorized models were established based on single-institution data in the training phase and tested on multicentric data in the validation phase. The study was approved by Ethical Board of Masaryk Memorial Cancer Institute (MMCI; approval No. 2016/2832/MOU) and by relevant Danish authorities as described previously [11].

### Patients and study population

The control group for HE4ren development consisted of 66 gynecologically and oncologically healthy women with various levels of glomerular filtration rate. The endometrial cancer population for dMI prediction model development consisted of MMCI training cohort and validation cohorts. A retrospective training cohort was represented by 120 EC patients treated at Masaryk Memorial Cancer Institute, Comprehensive Cancer Centre in Brno, Czech Republic, between October 2011 and March 2015. Validation cohorts consisted of 33 EC patients treated at MMCI from April 2015 to January 2016 and 346 EC patients enrolled in the Danish Endometrial Cancer Study (ENDOMET) conducted at 3 tertiary gynecologic oncology centres, namely Odense University Hospital, Rigshospitalet Copenhagen University Hospital and Aalborg University Hospital, between September 2009 and January 2012 [11] (Table 2).

**Table 2:** Characteristics of EC patients

		MMCI training		MMCI validation		ENDOMET validation	
		Any grade	G1-or-G2	Any grade	G1-or-G2	Any grade	G1-or-G2
**No. of patients**		120	98	33	28	346	273
**Age**	years	65 (60-72)	65 (60-71)	61 (53-73)	62 (52-72)	65 (59-74)	65 (58-73)
	< 60	27 (22.5)	24 (24.5)	16 (48.5)	13 (46.4)	102 (29.5)	83 (30.4)
	≥ 60	93 (77.5)	74 (75.5)	17 (51.5)	15 (53.6)	244 (70.5)	190 (69.6)
**FIGO stage**	AEH					17 (4.9)	0 (0.0)
	IA	60 (50.0)	55 (56.1)	22 (66.7)	20 (71.4)	197 (56.9)	174 (63.7)
	IB	28 (23.3)	22 (22.4)	8 (24.3)	6 (21.4)	39 (11.3)	35 (12.8)
	II	21 (17.5)	14 (14.2)	1 (3.0)	1 (3.6)	40 (11.7)	35 (12.8)
	IIIA	1 (0.8)	0 (0.0)	1 (3.0)	0 (0.0)	18 (5.2)	12 (4.4)
	IIIB	2 (1.7)	1 (1.0)			4 (1.2)	3 (1.1)
	IIIC	7 (5.8)	5 (5.1)			25 (7.2)	14 (5.1)
	IVA	0 (0.0)	0 (0.0)			2 (0.6)	0 (0.0)
	IVB	1 (0.8)	1 (1.0)			4 (1.2)	0 (0.0)
	TIS			1 (3.0)	1 (3.6)		
**Myometrial invasion**	< 50%	69 (57.5)	60 (61.2)	24 (72.7)	22 (78.6)	219 (63.2)	176 (64.5)
	≥ 50% (dMI)	51 (42.5)	38 (38.8)	9 (27.3)	6 (21.4)	127 (36.7)	97 (35.5)
**Grade**	1	54 (45.0)	54 (55.1)	16 (48.5)	16 (57.1)	207 (59.8)	207 (75.8)
	2	44 (36.7)	44 (44.9)	12 (36.4)	12 (42.9)	66 (19.1)	66 (24.2)
	3	20 (16.7)		5 (15.2)		25 (7.2)	
	Not grade	2 (1.7)				48 (13.9)	
**creatinine**	μmol/l	67 (59-78)	67 (59-80)	67 (55-85)	72 (61-89)	65 (58-72)	65 (58-72)
**eGFR**	ml/min/1.73 m^2^	84 (66-90)	84 (60-90)	90 (66-90)	84 (66-90)	84 (72-96)	84 (72-96)
**HE4**	pmol/l	62.6 (43.7-93.7)	60.5 (42.2-85.2)	52.3 (35.0-74.5)	43.6 (34.7-77.1)	60.7 (41.0-104.4)	60.4 (41.5-94.2)
**CA125**	U/ml	17.7 (12.0-30.2)	16.8 (11.6-30.0)	16.1 (11.7-21.4)	15.9 (11.5-20.4)	17.1 (9.5-28.1)	16.0 (9.3-25.6)
	< 35	97 (80.8)	81 (82.7)	29 (87.9)	26 (92.9)	282 (81.5)	230 (84.2)
	≥ 35	23 (19.2)	17 (17.3)	4 (12.1)	2 (7.1)	64 (18.5)	43 (15.8)

For categorical parameters, absolute numbers and percentage are shown in brackets. For continuous parameters, median and interquartile range are presented in brackets.

Histological grade was assessed from preoperative endometrial curettage specimens and determined according to WHO Classification of Tumours of Female Reproductive Organs. Hysterectomy specimens were evaluated by a dedicated gynecological pathologist perioperatively. The uterus external surface was examined macroscopically and the uterus dissected from cervix to fundus along its longitudinal axis. The myometrium was completely cut in parallel sections with the distance of 5 – 10mm. The deepest macroscopic tumor invasion and the corresponding uterus wall thickness were measured. One specimen from the deepest invasion site was examined perioperatively in frozen sections and histological type, grade and the deepest myometrial invasion in the form of x/y mm were reported to the operating room.

### Laboratory testing

Preoperative blood samples were obtained from EC patients within 2 weeks before scheduled surgical treatment. For CA125 and HE4 quantification, serum was analyzed within 2 hours from specimen sampling at MMCI, and aliquoted and stored at –80 °C until analysis in ENDOMET samples. CA125 and HE4 serum concentrations were measured by chemiluminescence microparticle immunoassay using an Architect i2000sr (Abbott) analyzer in all participating centers. In-house validation of HE4 measurement linearity ≥ 10 pmol/l have been performed (data not shown). Creatinine was measured in Li-heparin plasma by an enzymatic method with IDMS (isotopic dilution mass spectrometry) calibration using center-specific technology. Subsequently, estimated glomerular filtration rates (eGFR) were calculated by CKD-EPI equations based on sex, age and creatinine levels without race-adjustment [42].

### Statistical analysis

The relation between HE4 and eGFR was expressed using a piecewise linear regression model with logarithmically transformed (natural log) HE4 concentrations. Based on this relationship, a new mathematical parameter “HE4ren” was developed. Spearman correlation coefficient is presented for the association of HE4 or HE4ren with age. The association of the considered variables with dMI was assessed by the univariable analysis, using the Pearson chi-square test and the Fisher exact test for categorical data and the Mann-Whitney test for continuous data. Factors showing predictive value in the univariable analysis were evaluated by the multivariable analysis. A binary logistic regression with backward elimination method was used to construct a model to assess the probability of having dMI. Results are presented by their odds ratios (OR) with 95% confidence interval (CI). The significance level used for a variable to remain in the model was 0.1. Models considering continuous variables were developed with both log-transformed HE4 and log-transformed HE4ren. Continuous parameters were categorized where cut-offs were determined using ROC analysis. One model with continuous parameters and one with categorical parameters were selected and evaluated in more detail for use in clinical practice. To make the model more user-friendly, a score number was assigned to each parameter as the corresponding coefficient from the multivariable regression model multiplied by 10. Statistical analyses were performed using R version 3.2.4.

## SUPPLEMENTARY MATERIALS FIGURES AND TABLES



## Abbreviations

AUC: area under curve
CA125: cancer antigen 125
CI: confidence interval
CKD: chronic kidney disease
CKD-EPI: chronic kidney disease epidemiology collaboration
dMI: deep myometrial invasion
EC: endometrial cancer
eGFR: estimated glomerular filtration rates
ENDOMET: the Danish Endometrial Cancer Study
FIGO: International Federation of Gynecology and Obstetrics
G: grade
HE4: Human epididymis protein 4
HE4ren: HE4 level adjusted to glomerular filtration rate
IDMS: isotopic dilution mass spectrometry
IQR: interquartile range
MI: myometrial invasion
MMCI: Masaryk Memorial Cancer Institute
NA: not available
No.: number
NPV: negative predictive value
NS: not significant
OR: odds ratio
PPV: positive predictive value
ROC: receiver operation characteristic
Se: sensitivity
Sp: specificity
WHO: World Health Organisation
