# Supplementary material for: Adjustment of serum HE4 to reduced glomerular filtration and its use in biomarker-based prediction of deep myometrial invasion in endometrial cancer
Source: Oncotarget. 2017 Nov 21;8(64):108213–22. doi: 10.18632/oncotarget.22599 (PMC5746137; doi:10.18632/oncotarget.22599)
Supplement: Supplementary file 1 [file oncotarget-08-108213-s001.pdf]

Adjustment of serum HE4 to reduced glomerular filtration and its use in biomarker-based prediction of deep myometrial invasion in endometrial cancer

SUPPLEMENTARY MATERIALS

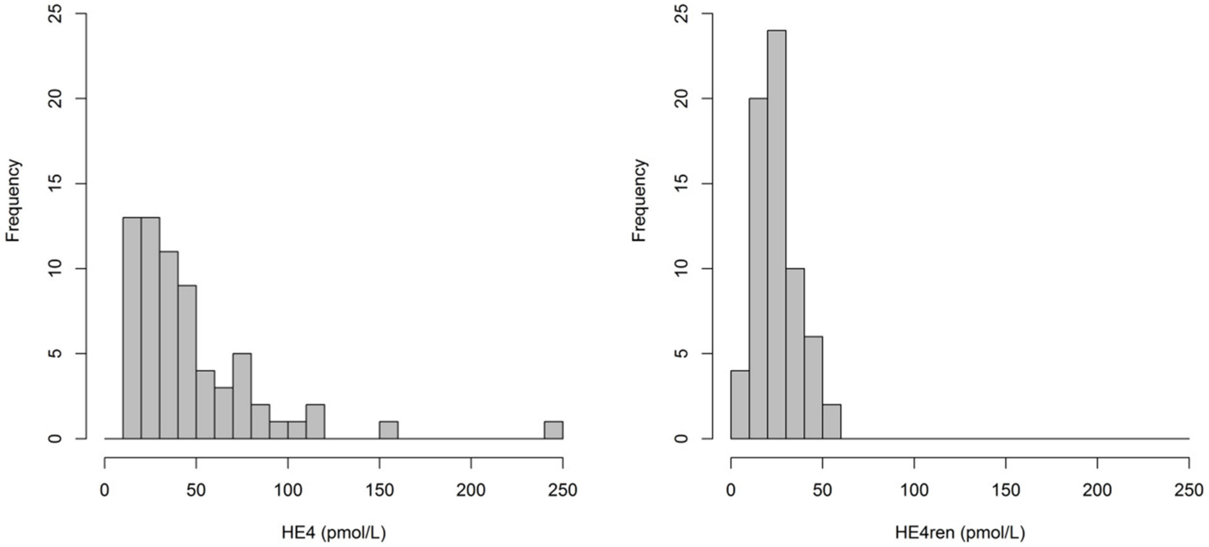

Supplementary Figure 1: Distribution of HE4 (left) and HE4ren (right) in gynecologically and oncologically healthy control group including individuals with reduced glomerular functions. Median age was 64.5 years (IQR 51-74). Number of individuals younger than 60 years was 28 (42.4%). Median eGFR was 74 ml/min/1.73 m^2 (IQR 52-86). Median serum level of HE4 was 34.5 pmol/l (IQR 22.3-58.3).

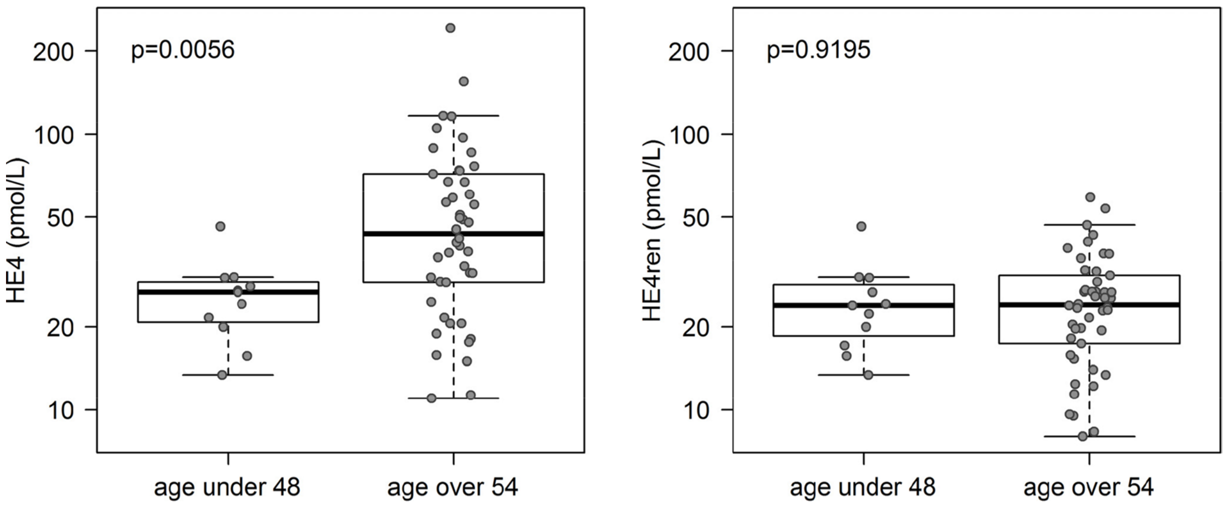

Supplementary Figure 2: HE4 (left) and HE4ren (right) in the context of menopausal status. Menopausal status was defined by age using conservative proxy with women under 48 years considered as premenopausal and over 54 years considered as postmenopausal. Median eGFR was 106 ml/min/1.73 m^2 (IQR 90 - 109) for individuals under 48 years and 57 ml/min/1.73 m^2 (IQR 48-77) for individuals over 54 years.

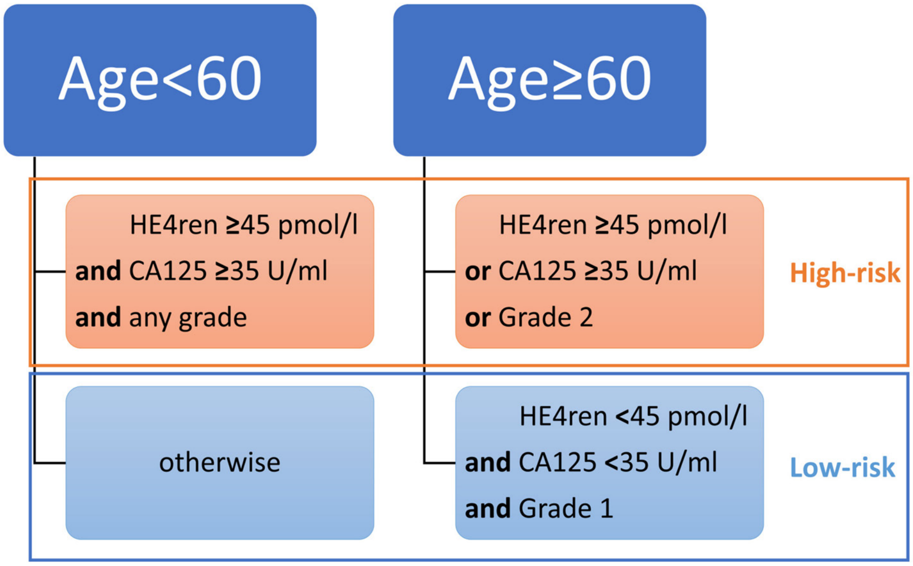

Supplementary Figure 3: The algorithm tree demonstrating the simplified model for preoperative dMI prediction in G1/2 patients.

Supplementary Table 1: HE4ren distribution in the training and validation EC datasets. Median and interquartile ranges are shown in brackets

	MMCI training		MMCI validation		ENDOMET validation	
	Any grade	G1+G2	Any grade	G1+G2	Any grade	G1+G2
HE4ren, pmol/l	46.6 (32.6-67.1)	43.6 (31.7-62.6)	40.1 (30.6-55.0)	36.9 (30.4-49.7)	49.4 (34.4-82.4)	49.5 (35.3-79.3)

Supplementary Table 2 : Univariable analysis for prediction of dMI. NA - Not Available, low number of patients for analysis in the MMCI validation

		MMCI training				MMCI validation				ENDOMET validation			
		Any grade		G1-or-G2		Any grade		G1-or-G2		Any grade		G1-or-G2	
		AUC	p-value	AUC	p-value	AUC	p-value	AUC	p-value	AUC	p-value	AUC	p-value
**Age**	years	0.6962	0.0002	0.6963	0.0011	0.5718	0.5436	0.6216	0.3845	0.5694	0.0314	0.5569	0.1197
	Cut-off 60		0.0009		0.0032		>0.9999		0.6546		0.0023		0.0406
**Grade**	G1 vs G2 vs G3		0.0583		0.1518		NA		NA		0.0003		0.0004
**CA125**	U/ml	0.6682	0.0017	0.6548	0.0102	0.7778	0.0142	0.7803	0.0386	0.7091	<0.0001	0.7022	<0.0001
	Cut-off 35		0.0009		0.0052		0.0031		0.0397		<0.0001		<0.0001
**HE4**	pmol/l	0.7951	<0.0001	0.7912	<0.0001	0.7593	0.0229	0.7424	0.0775	0.7086	<0.0001	0.7069	<0.0001
**HE4ren**	pmol/l	0.7783	<0.0001	0.7890	<0.0001	0.8241	0.0036	0.8030	0.0240	0.7201	<0.0001	0.7226	<0.0001

Supplementary Table 3: Calculation of the probability of having myometrial invasion ≥ 50% using the continuous models

Model	Formula of P(dMI)
**Any grade**	
Continuous-all-HE4	$P(dMI)=\frac{1}{1+e^{11.8-0.6age-1.8\log HE4}}$
Continuous-all-HE4ren	$P(dMI)=\frac{1}{1+e^{13.7-0.1age-1.9\log HE4ren}}$
**G1-or-G2**	
Continuous-G1/2-HE4	$P(dMI)=\frac{1}{1+e^{11.8-0.1age-1.6\log HE4-0.8(grade-1)}}$
**Continuous-G1/2-HE4ren**	$P(dMI)=\frac{1}{1+e^{15.2-0.1age-2.1\log HE4ren-0.9(grade-1)}}$

Supplementary Table 4: Classification table of analytical parameters of the final continuous G1/2 HE4ren model

Cut-off	MMCI training				MMCI validation				ENDOMET validation			
	Se (%)	Sp (%)	PPV (%)	NPV (%)	Se (%)	Sp (%)	PPV (%)	NPV (%)	Se (%)	Sp (%)	PPV (%)	NPV (%)
10%	97.4	28.3	46.3	94.4	83.3	50.0	31.3	91.6	91.8	26.1	40.6	85.2
20%	89.5	48.3	52.3	87.9	66.7	59.1	30.8	86.7	79.4	43.8	43.8	79.4
30%	84.2	66.7	61.5	87.0	66.7	68.2	36.4	82.2	72.2	55.7	47.3	78.4
40%	78.9	78.3	69.7	85.5	66.7	86.4	57.1	90.5	60.8	65.9	49.6	75.3
**43% ***	**78.9**	**85.0**	**76.9**	**86.4**	**66.7**	**86.4**	**57.1**	**90.5**	**59.8**	**70.5**	**52.7**	**76.1**

Se = Sensitivity, Sp = Specificity, PPV = positive predictive value, NPV = negative predictive value, *optimal cut-off for the MMCI training dataset. The cut-off value confirmed by validation sets is shown in bold.

Supplementary Table 5: Categorical model development and validation. Multivariable analysis of dMI prediction with categorical variables including simple categorical score development

Model	Predictor				AUC		
	**Age (≥ 60 vs < 60)**	**HE4ren (≥ 45 vs < 45)***	**Grade**	**CA125 (≥ 35 vs < 35)***	**MMCI training**	**MMCI validation**	**ENDOMET validation**
**Any grade**	OR: 6.02 (1.82-25.4) p = 0.0064	OR: 4.88 (2.06-12.2) p = 0.0005	NS	OR: 3.94 (1.33-13.2) p = 0.0172	0.7801	0.7338	0.7219
**G1-or-G2 full**	**OR: 8.24 (2.01-49.3) p = 0.0083**	**OR: 5.95 (2.21-17.5) p = 0.0006**	**OR: 2.37 (0.88-6.71) p = 0.0923**	**OR: 4.17 (1.17-17.7) p = 0.0352**	**0.8186**	**0.7803**	**0.7249**

$$P(dMI) = \frac{1}{1+e^{3.8-2.1age_k-1.8HE4ren_k-1.4Ca125_k-0.9(grade-1)}}$$

Model	Specific score values for categorical variables				probability of dMI with total score			
**G1-or-G2 score**	Age ≥ 60	HE4ren ≥ 45 pmol/l	Grade 2	CA125 ≥ 35 U/ml	*P*	Score	*P*	Score
					2.2	0	40	33.9
					10	16.0	50	38.0
	21.1	17.8	8.6	14.3	20	24.1	60	42.0
					25	27.1	70	46.4
					30	29.5	80	51.8
					33	30.9	90	59.9
**G1-or-G2 score simple**	**3**	**2**	**1**	**2**	**Low-risk**	**≤ 3**	**High-risk**	**≥ 4**

NS = nonsignificant p > 0.1. The final model with real-life clinical applicability is shown in bold with detailed description of the model where P(dMI) stands for the probability of having myometrial invasion ≥ 50%, age_k=1 if patient is 60 years old or more (otherwise age_k = 0), $HE4ren_k$ = 1 if HE4ren is ≥ 45 pmol/l (otherwise $HE4ren_k$ = 0), $CA125_k$ = 1 if CA125 is ≥ 35 U/ml (otherwise $CA125_k$ = 0). The probability of dMI as a function of total score is shown for various levels of probability. *Cut-off values were calculated from ROC analysis and cut-offs with optimal sensitivity and specificity were further exploited for age and HE4ren (data not shown). In the case of CA125, ROC analysis revealed optimal cut-off values of 24.5 U/ml for any-grade patients and 26.0 U/ml for G1-or-G2 patients (data not shown). Thus, CA125 cut-off values 20 U/ml, 25 U/ml and 35 U/ml were tested in categorical models with optimal analytical efficacy for CA125 cut-off 35 U/ml (data not shown).

Supplementary Table 6: Classification table of analytical parameters of the final categorical G1/2 HE4ren model

Cut-off	MMCI training				MMCI validation				ENDOMET validation			
	Se (%)	Sp (%)	PPV (%)	NPV (%)	Se (%)	Sp (%)	PPV (%)	NPV (%)	Se (%)	Sp (%)	PPV (%)	NPV (%)
10%	100	26.6	46.3	100	83.3	45.5	29.4	90.9	92.8	18.8	38.6	82.5
**25%**	**89.5**	**56.6**	**56.6**	**89.5**	**66.7**	**77.3**	**44.4**	**89.5**	**77.3**	**59.7**	**51.4**	**82.7**
33% *	76.3	71.7	63.0	82.7	50.0	86.4	50.0	86.4	72.2	64.2	61.9	80.7

Se = Sensitivity, Sp = Specificity, PPV = positive predictive value, NPV = negative predictive value, *optimal cut-off for MMCI training dataset. The cut-off value confirmed by validation sets is shown in bold.
